# Case Report: T‐Lymphoblastic Leukemia in a Dog With Erythrophagia

**DOI:** 10.1111/vcp.70117

**Published:** 2026-07-22

**Authors:** Francisca B. Lang, Matheus Y. dos Santos, Vinicius N. Hirata, Felipe A. B. de Sousa, Augusto de Oliveira, Lucas T. de Castro, Rafael A. Fighera, Alexandre Krause

**Affiliations:** ^1^ Veterinary Clinical Pathology Laboratory, University Veterinary Hospital Federal University of Santa Maria Santa Maria Rio Grande do Sul Brazil; ^2^ Veterinary Pathology Laboratory Federal University of Santa Maria Santa Maria Rio Grande do Sul Brazil; ^3^ Veterinary Pathology Section, Veterinary School Federal University of Rio Grande do Sul Porto Alegre Rio Grande do Sul Brazil

**Keywords:** canine, erythrophagia, immunohistochemistry, neoplasia

## Abstract

A 2‐year‐old male German Shepherd dog was presented with progressive weight loss, lethargy, and hyporexia. Hematologic analysis revealed a moderate macrocytic normochromic anemia and moderate thrombocytopenia. Additionally, a pronounced leukocytosis was observed, characterized by 33% large lymphoid cells, marked neutrophilia with a significant left shift, and intense lymphocytosis and monocytosis. These large lymphoid cells exhibited moderately basophilic cytoplasm and nuclei with chromatin loss and occasional prominent nucleoli, along with erythrophagia and both typical and atypical mitotic figures. Serum chemistry showed mild hypoproteinemia and hypoalbuminemia. Bone marrow aspirate revealed high cellularity with lymphoid predominance and 40.6% blasts per 1000 nucleated cells, of which 2.6% showed erythrophagia. PCR for antigen receptor rearrangements performed on DNA extracted from the bone marrow smear confirmed a clonal T‐cell receptor rearrangement. Despite supportive treatment and initial chemotherapy, no clinical recovery was observed, and the patient was euthanized 8 days post‐diagnosis. Necropsy revealed marked splenohepatomegaly, intensely red bone marrow, mesenteric lymphadenomegaly, gastric and duodenal ulcers, and jejunal intussusception. Histologically, the bone marrow was hypercellular with monomorphic neoplastic blast cells, some exhibiting erythrophagia. Neoplastic infiltration was observed in the liver, spleen, lymph nodes, and adrenal glands. Immunohistochemistry showed blast cells positive for CD3 and negative for CD20. The final diagnosis was T‐lymphoblastic leukemia with erythrophagia, underscoring the diagnostic importance of hematologic, histopathologic, and immunohistochemical evaluations in diseases with nonspecific clinical signs. Furthermore, the uncommon presentation of erythrophagia by neoplastic cells may be associated with severe anemia and poor prognosis in this patient.

## 1 Case Report

An intact, 2‐year‐old male German Shepherd dog was presented with nonspecific clinical signs, including progressive weight loss, lethargy, and hyporexia. Physical examination revealed pale mucous membranes and mild dehydration. Serum biochemistry (Day 0) revealed hypoproteinemia (4.7 g/dL; reference interval [RI]: 5.4–7.4 g/dL) and hypoalbuminemia (2.15 g/dL; RI: 2.7–4.5 g/dL). The CBC showed moderate thrombocytopenia and macrocytic normochromic anemia. Moderate anisocytosis, mild polychromasia, and a small number of spherocytes were observed, along with mildly icteric plasma. Reticulocyte counting was not performed. Additionally, pronounced leukocytosis was observed (Figure 1A), characterized by 33% large lymphoid cells, marked neutrophilia with a significant left shift, and intense lymphocytosis and monocytosis (Table 1). Reactive lymphocytes were also identified. The large lymphoid cells (10–14 μm) had moderately basophilic cytoplasm and nuclei containing loose chromatin and occasional prominent nucleoli. Additionally, both typical and atypical mitotic figures (Figure 1B) and cytoplasmic fragments (Figure 1C) were observed. Rare erythrophagia (0.6% of nucleated cells) was also noted, characterized by large lymphoid cells engulfing erythrocytes (Figure 1D). Five days after the initial presentation, a follow‐up CBC was performed, revealing a worsening of the anemia, with persistent leukocytosis and thrombocytopenia (Table 1).

**FIGURE 1:**
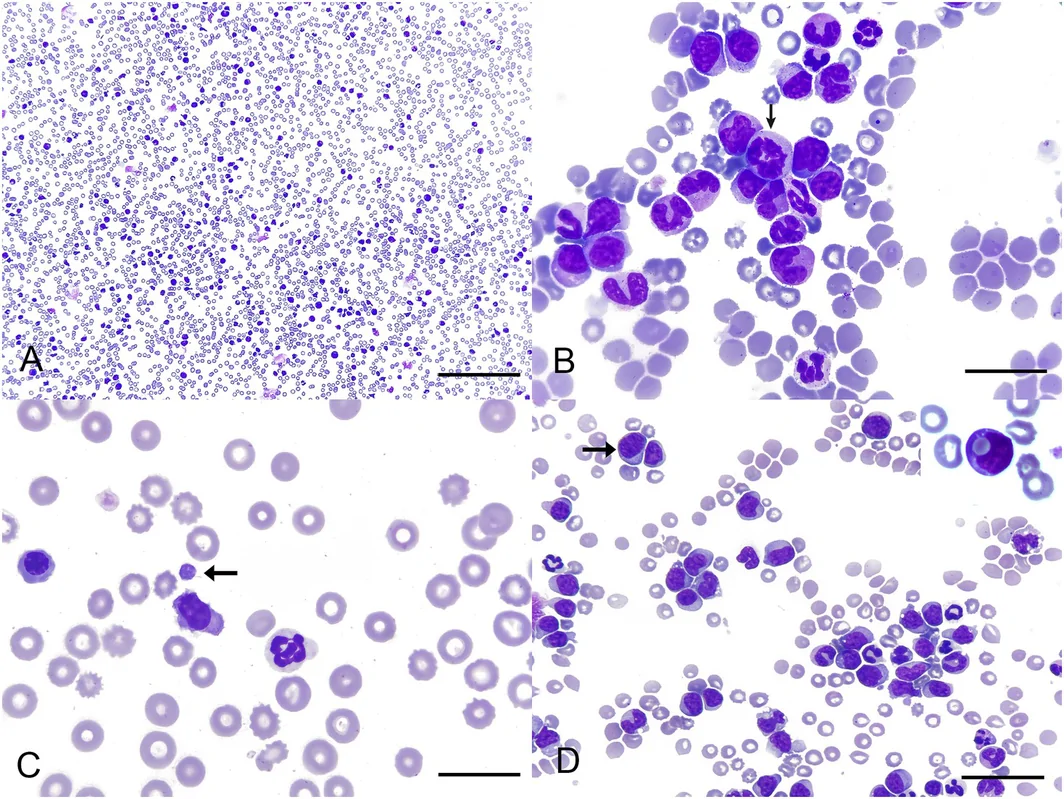
Blood film from a dog with T‐lymphoblastic leukemia. (A) Marked leukocytosis characterized by the presence of large lymphocytes and neutrophils. 10× objective, scale bar, 200 μm. (B) Mitotic figure (black arrow) 100× objective, scale bar 20 μm. (C) Cytoplasmic fragments were frequently observed (black arrow). Scale bar 20 μm. (D) Presence of large lymphoid cells (black arrow). Scale bar 40 μm. Inset: Erythrophagia, ×100 objective. Romanowsky aqueous stain.

**TABLE 1:** Complete blood count results at presentation and 5 days later in a 2‐year‐old German Shepherd dog with T‐cell lymphoblastic leukemia.

	CBC (Day 0)	CBC (Day 5)	Reference values [1]
RBC (×10^12^/L)	2.35	1.96	5.5–8.5
HGB (g/dL)	7.1	5.8	12–18
Packed cell volume (%)	20.0	15.9	37–55
MCV (fL)	85.0	81.0	60–72
MCHC (g/dL)	35.5	36.4	33–38
Nucleated RBCs (×10^9^/L)	9	3.4	0
Platelets (×10^9^/L)	64	108	200–500
WBC (×10^9^/L)	113.2	115.7	6–17
Large lymphoid cells (×10^9^/L)	37.4	11.6	0
Bands neutrophils (×10^9^/L)	6.8	2.3	0–0.3
Segmented neutrophils (×10^9^/L)	36.2	35.9	3–11.5
Small lymphocytes cells (×10^9^/L)	14.7	46.3	1–4.8
Monocytes (×10^9^/L)	18.1	18.5	1–1.3
Eosinophils (×10^9^/L)	0	1.2	1–1.2

Bone marrow analysis, obtained by iliac crest aspiration on the same day the second CBC was performed, showed markedly increased cellularity (85%–95%), in comparison with adipose tissue (Figure 2A). Megakaryocyte counts per spicule ranged from 5 to 17, observed using the 10× objective, and the myeloid‐to‐erythroid (M:E) ratio was estimated at 2.2. The erythroid lineage was mildly hypoplastic, with complete and well‐staged maturation. The myeloid series was mildly hyperplastic and showed complete maturation; nevertheless, depletion of mature neutrophils and a mild left shift were observed. Occasionally, dysplastic cells, such as giant and ring‐shaped neutrophils, were seen (Figure 2B). A predominant homogeneous population of lymphoid‐like cells, consistent with neoplastic blasts, accounted for 40.6% of the 1.000‐cell differential. These blasts shared morphologic features with those large lymphoid cells observed in peripheral blood but were larger in size (16–20 μm). A high frequency of both typical and atypical mitotic figures and cytoplasmic fragments was observed. An interesting finding was the recurrent presence of erythrocytes engulfed by lymphoid‐like cells (2.6% of nucleated cells) (Figure 2C). Based on the CBC and bone marrow analysis, a diagnosis of acute leukemia was established.

**FIGURE 2:**
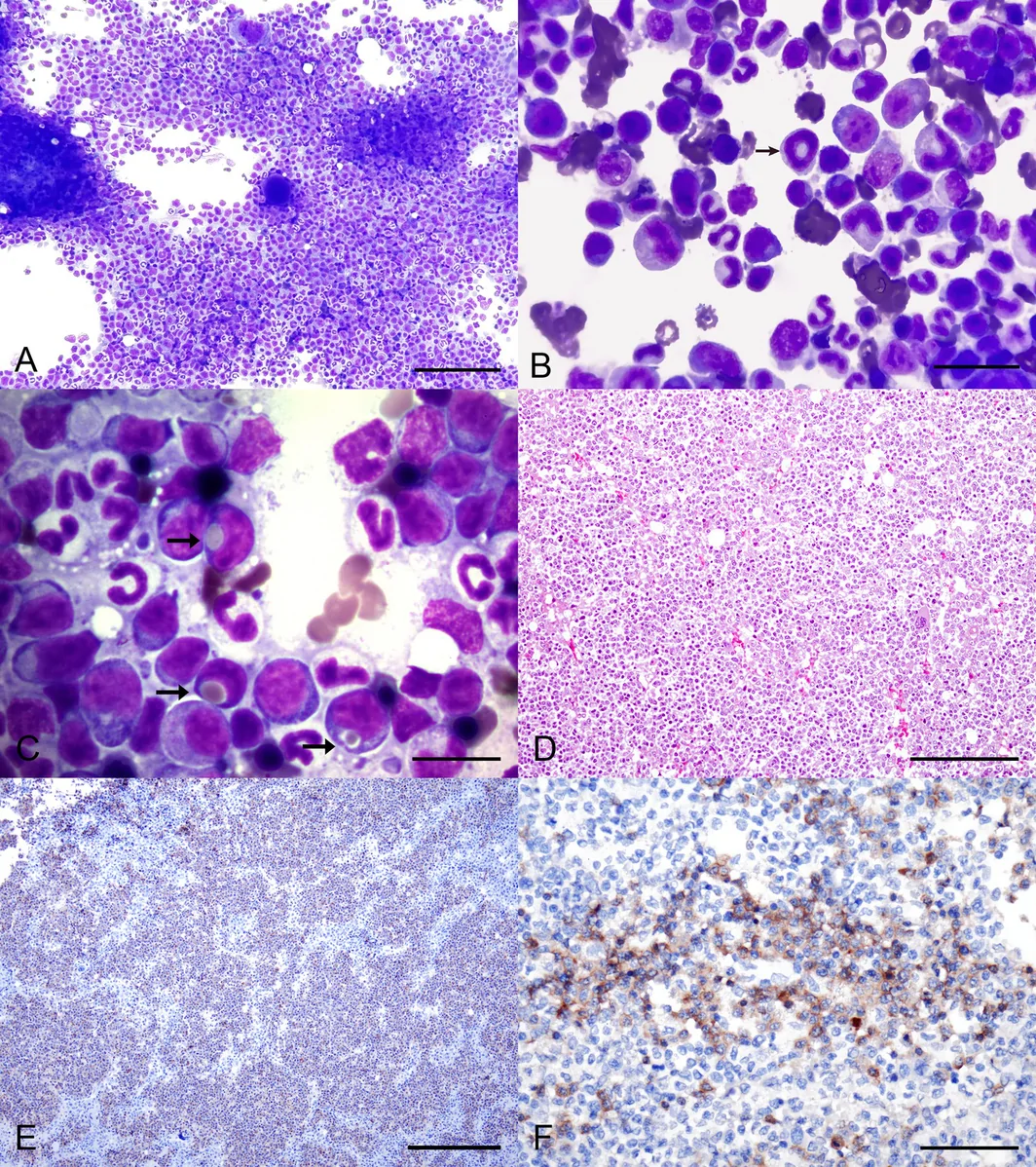
Bone marrow cytology from a dog with T‐lymphoblastic leukemia. (A) Myelogram presents a monomorphic population of neoplastic cells replacing normal hematopoietic population. 20× objective, scale bar 100 μm. (B) Cytology demonstrates dysplastic changes, with an increased proportion of band neutrophils relative to segmented neutrophils. A dysplastic ‘donut‐shaped’ neutrophil (black arrow) is visible. Scale bar 20 μm. (C) Erythrophagia figures (black arrow). Romanowsky aqueous stain, Scale bar 20 μm. (D) Histopathologic section. Marked cellularity with replacement of normal hematopoietic tissue by numerous round cells exhibiting a high nucleus‐to‐cytoplasm ratio. These cells display blast‐like features, including loose chromatin and one or more prominent nucleoli. Few adipocytes and megakaryocytes are present, along with a small number of myeloid and erythroid precursors. H&E stain, Scale bar 200 μm. (E) Immunohistochemical staining of bone marrow. Extensive replacement of marrow tissue by neoplastic cells, with strong cytoplasmic CD3 positivity in 80%–90% of cells. Scale bar 200 μm. (F) Detail of cytoplasmic CD3 positivity in neoplastic cells, marked in brown. Scale bar 300 μm. The chromogen used was Romulin AEC (BioCare), and slides were counterstained with hematoxylin.

Bone marrow smear slides were submitted for PCR‐antigen receptor rearrangements (PARR), which revealed clonality for the TCRγ gene (T‐cell receptor gamma) and a polyclonal pattern for the IGH gene (immunoglobulin heavy chain). These findings narrowed the differential diagnoses to T‐cell lymphoblastic leukemia (T‐LL) and acute myeloid leukemia (AML) [2].

Based on laboratory findings and the clinical presentation, supportive therapy was initiated, including the administration of ondansetron (1 mg/kg IV TID), omeprazole (1 mg/kg IV SID), metamizole (25 mg/kg IV BID), and metronidazole (15 mg/kg IV BID). Additionally, prednisolone therapy (2 mg/kg PO BID) was initiated for 8 days, and a single dose of vincristine sulfate (0.75 mg/m^2^ IV) was administered 3 days after diagnosis; a packed red blood cell transfusion was performed on day five after diagnosis.

Due to rapid clinical deterioration without any response to therapy, the patient was euthanized on day 8 after diagnosis. Necropsy findings included cachexia, markedly pale oral and ocular mucous membranes, and pasty, dark reddish‐black feces. The spleen and liver were diffusely and markedly enlarged. Mesenteric lymph nodes were also markedly enlarged, with a brownish coloration and a loss of corticomedullary distinction on section. The femoral bone marrow appeared intensely red and filled the entire medullary canal. In the jejunal portion of the small intestine, a focally extensive area of intussusception was identified; the intussuscepted segment was markedly dark red. The gastric mucosa showed multifocal areas of superficial erosions.

Histopathological examination of the bone marrow revealed severe replacement of normal hematopoietic elements by a monomorphic population of large lymphoid cells (Figure 2D). These cells had large, round to oval nuclei with loose chromatin, prominent nucleoli, and scant basophilic cytoplasm. Rare cells exhibiting erythrophagia were also observed. High proliferative activity was evident, as indicated by numerous mitotic figures, with 16 mitotic figures observed within an area of 2.37 mm^2^ (FN22/10 HPF). Infiltration by the same neoplastic cell population was identified in the liver, spleen, mesenteric lymph nodes, and adrenal glands.

Immunohistochemical analysis of the bone marrow revealed strong cytoplasmic positivity for CD3 (polyclonal antibody, rabbit origin, ready‐to‐use, Dako) in the neoplastic blasts. No positivity for CD20 (polyclonal antibody, rabbit origin, 1:400 dilution, Invitrogen) was observed in the neoplastic cells. Romulin AEC (BioCare) was used as the chromogen, and slides were counterstained with hematoxylin. Diffuse replacement of the bone marrow architecture by neoplastic cells was observed, with approximately 70% of the total cellularity showing positive CD3 expression, confirming the T‐cell‐lineage phenotype of the neoplastic population (Figure 2E,F).

## 2 Discussion

Based on the hematologic, histologic, and immunohistochemical findings, a diagnosis of T‐LL was established [3]. Acute lymphoblastic leukemia (ALL) originates from neoplastic lymphoblasts and/or prolymphocytes present in the bone marrow, which are often also found in peripheral blood [2]. It is considered one of the most aggressive neoplasms in companion animals [3]. It has a poor prognosis, with a median survival time ranging from 8 to 55 days, even with chemotherapy treatment [4, 5].

The clinical signs of the patient were nonspecific, as commonly reported in cases of acute leukemia (AL) [4, 6, 7]; however, in this case, some of the findings may also be correlated with the presence of gastric ulcers and intussusception. For the diagnosis of AL, both the CBC and the microscopic evaluation of the blood and bone marrow smears proved to be indispensable tools for guiding diagnostic interpretation. Considering that the mean age of dogs diagnosed with AL ranges between 7.5 and 7.9 years [5, 7], other conditions are typically considered more likely differential diagnoses.

In the present case, the CBC revealed thrombocytopenia, macrocytic normochromic anemia, marked leukocytosis, and a substantial number of large lymphoid cells, which were key findings to support the diagnosis of AL [7, 8]. The thrombocytopenia observed in peripheral blood may be primarily a consequence of peripheral destruction or consumption rather than a direct effect of the leukemic cells. Myelophthisis as a cause of anemia could not be ruled out, given the presence of a highly cellular lymphoid population in the bone marrow, even though the hematopoietic lineages were still present in the aspirate (slight myeloid hyperplasia, mild megakaryocytic hyperplasia, mild erythroid hypoplasia, and an M:E ratio of 2.2). Cancer‐associated inflammation could also contribute to anemia, due to the inhibitory effects of inflammatory cytokines on erythropoiesis [7, 9]. In addition, inflammation has a stimulatory effect on platelet production (IL‐6 stimulates TPO by the liver) and would also explain the bone marrow megakaryocytic hyperplasia observed [10]. An immune‐mediated hemolytic process may be considered, based on the presence of spherocytes and mildly icteric plasma, as a contributing cause of the anemia. However, supportive tests, such as the saline agglutination test and the direct Coombs' test, were not performed. Although a causal association between neoplasia and immune‐mediated hemolytic anemia has not been clearly established in the veterinary literature, neoplasia cannot be excluded as a potential cause of the suspected hemolysis [11]. Macrocytosis is associated with a regenerative erythropoiesis process or the occurrence of dyserythropoiesis [2] (which was not found in this case); however, an additional method for better evaluation of erythropoiesis would be to perform a reticulocyte count (not performed).

Intense leukocytosis, with a left shift and monocytosis seen in peripheral blood, could be explained by alterations in the bone marrow microenvironment caused by tumor cell invasion and proliferation. Cytokines released by tumor cells and affected stroma are part of the well‐known mechanism of tumor‐associated inflammation [2]. A corresponding feature was observed in bone marrow aspirate (mild myeloid hyperplasia).

The bone marrow aspirate confirmed the diagnosis of acute leukemia ante‐mortem by demonstrating high cellularity and blast counts exceeding 20% (40.6%). Furthermore, neoplastic cells with lymphoid morphology were observed engulfing red blood cells (erythrophagia). This phenomenon was previously described in other neoplasms (hemangiosarcomas, hemophagocytic histiocytic sarcomas, osteosarcomas, mast cell tumors, and T‐cell lymphomas) and is characterized by an intercellular interaction referred to as xenocannibalism, since the term “erythrophagocytosis” is appropriately applied only to the so‐called professional phagocytes [12, 13, 14]. To our knowledge, this is the first report of erythrophagocytic T‐lymphoblastic leukemia in a dog [13, 15]. The hypothesis of hemophagocytic syndrome was ruled out, as fewer than 2% of macrophages exhibiting hemophagocytosis were observed in the bone marrow aspirate [16].

Flow cytometry, the gold standard for immunophenotypic characterization of leukemias, was not performed because this laboratory analysis was unavailable in the region. In this context, flow cytometry would have improved immunophenotypic characterization by enabling the determination of neoplastic cell maturation and the application of objective lineage‐assignment criteria to confirm lymphoid origin and exclude myeloid phenotypes, using fresh bone marrow or peripheral blood samples [17]. As an alternative, bone marrow smear slides were submitted for PARR analysis, which demonstrated clonality for T‐cell receptors. However, this result alone is not confirmatory for T‐LL [18], additional tests would be required for a more accurate diagnosis.

Despite supportive treatment, antibiotic therapy, glucocorticoids, and chemotherapy, the patient's survival was only 8 days. Necropsy revealed ulcerative lesions in the stomach and duodenum. In human patients with T‐cell lymphoma/leukemia, gastric lesions were observed in 30.3% of cases [19]. The cause of the jejunal intussusception remains unknown in this case. The presence of leukemic lymphocytes in the liver, spleen, mesenteric lymph nodes, and adrenal glands highlights the disseminative and invasive behavior characteristic of ALL [20].

The absence of superficial peripheral lymphadenomegaly, combined with the marked hematologic abnormalities (anemia, thrombocytopenia, and expansion of large lymphoid cells in circulation), made stage V lymphoma less likely as a differential diagnosis [21, 22]. Although the diagnosis was T‐LL, it is important to emphasize that the prognosis remains guarded for all forms of acute leukemia in dogs, with an average survival time of approximately 8 days for B‐cell acute leukemia, 10 days for T‐LL [5, 6]. Additionally, the presence of erythrophagic figures within the neoplastic cells is a rare event and can contribute to the poor prognosis, as erythrophagia has been associated with progressive deterioration of the anemic state [12, 23].

In conclusion, the patient in this report, a young German Shepherd dog, was diagnosed with acute T‐LL exhibiting erythrophagia. The diagnosis was established through an integrated evaluation of the CBC, bone marrow aspirate, necropsy findings, histopathology, PARR, and immunohistochemistry. This case highlights the importance of a combined diagnostic approach and describes a very uncommon feature: erythrophagia of neoplastic cells. This uncommon finding may likewise be associated with the grave prognosis and marked anemia documented in this patient.

## Funding

This work was supported by Coordenação de Aperfeiçoamento de Pessoal de Nível Superior, 0001. Conselho Nacional de Desenvolvimento Científico e Tecnológico.

## Conflicts of Interest

The authors declare no conflicts of interest.

## Data Availability

Data sharing not applicable to this article as no datasets were generated or analysed during the current study.
